# Mental and Physical Health in Swiss Older Survivors of Enforced Child Welfare Practices

**DOI:** 10.1093/geroni/igab046.2079

**Published:** 2021-12-17

**Authors:** Myriam Thoma, Florence Bernays, Carla Eising, Andreas Maercker, Shauna Rohner

**Affiliations:** 1 University of Zurich, Zurich, Zurich, Switzerland; 2 University of Zürich, University of Zurich, Zurich, Switzerland; 3 University of Zürich, Zurich, Zurich, Switzerland; 4 University of Zurich, University of Zürich, Zurich, Switzerland; 5 University of Zurich, Zürich, Zurich, Switzerland

## Abstract

It is the purpose of child welfare practices to provide a protective environment for minors. However, welfare practices for children and adolescents have also been linked to a higher risk for maltreatment, trauma, and deprivation. Due to such early-life adversity, affected individuals often report a life course depicted by further trauma, socio-economic disadvantage, mental and physical ill-health. Examination of the long-term health correlates of enforced child welfare practices, as well as potential mediators, have previously been neglected in later life. It was therefore the purpose of these studies to examine the long-term correlates of enforced child welfare practices; the associated maltreatment, trauma, and deprivation; and the physical and mental health outcomes in Swiss older survivors (n=132, MAGE=71 years) and an age-matched control group (n=125). These studies further examined the mediating role of socio-economic factors (e.g., education, income), self-esteem, and self-compassion. Mental health was assessed with a structured clinical interview; physical health, self-esteem, and self-compassion with psychometric instruments. Survivors reported significantly more types and severity of childhood maltreatment, trauma, and deprivation than the control group. They also reported significantly more lifetime and current mental health disorders and more physical illnesses. Socio-economic factors and self-esteem, but not self-compassion, acted as significant mediators. Exposure to maltreatment, trauma, and deprivation in childhood and adolescence is linked to poorer mental and physical health in later life. Potential targets for intervention and health-protective measures include socio-economic factors and self-esteem, which were found to diminish the detrimental long-term impact of early-life adversity and disadvantage into later life.

